# Performance of Cryptococcal Antigen Lateral Flow Assay Using Saliva in Ugandans with CD4 <100

**DOI:** 10.1371/journal.pone.0103156

**Published:** 2014-07-31

**Authors:** Richard Kwizera, Joyce Nguna, Agnes Kiragga, Jesca Nakavuma, Radha Rajasingham, David R. Boulware, David B. Meya

**Affiliations:** 1 Infectious Diseases Institute, Makerere University, Kampala, Uganda; 2 College of Veterinary Medicine, Animal Resource and Biosecurity, Makerere University, Kampala, Uganda; 3 Division of Infectious Diseases and International Medicine, Department of Medicine, University of Minnesota, Minneapolis, Minnesota, United States of America; 4 Makerere University College of Health Sciences, School of Medicine, Department of Medicine, Kampala, Uganda; University of Birmingham, United Kingdom

## Abstract

**Background:**

Cryptococcal meningitis can best be diagnosed by cerebrospinal fluid India ink microscopy, cryptococcal antigen detection, or culture. These require invasive lumbar punctures. The utility of cryptococcal antigen detection in saliva is unknown. We evaluated the diagnostic performance of the point-of-care cryptococcal antigen lateral flow assay (CrAg LFA) in saliva.

**Methods:**

We screened HIV-infected, antiretroviral therapy naïve persons with symptomatic meningitis (n = 130) and asymptomatic persons with CD4+<100 cells/µL entering into HIV care (n = 399) in Kampala, Uganda. The diagnostic performance of testing saliva was compared to serum/plasma cryptococcal antigen as the reference standard.

**Results:**

The saliva lateral flow assay performance was overall more sensitive in symptomatic patients (88%) than in asymptomatic patients (27%). The specificity of saliva lateral flow assay was excellent at 97.8% in the symptomatic patients and 100% in asymptomatic patients. The degree of accuracy of saliva in diagnosing cryptococcosis and the level of agreement between the two sample types was better in symptomatic patients (C-statistic 92.9, κ-0.82) than in asymptomatic patients (C-statistic 63.5, κ-0.41). Persons with false negative salvia CrAg tests had lower levels of peripheral blood CrAg titers (P<0.001).

**Conclusion:**

There was poor diagnostic performance in testing saliva for cryptococcal antigen, particularly among asymptomatic persons screened for preemptive treatment of cryptococcosis.

## Introduction

Cryptococcal meningitis is the most common cause of adult meningitis in Africa [1], and results in approximately 20–25% of AIDS-related deaths. [2]–[4]. The majority of cases occur in sub-Saharan Africa with an estimated 6-month survival of 20–60% [4]–[6]. Availability of early initiation of antiretroviral therapy (ART) in high-income countries has significantly reduced the burden of cryptococcal meningitis, yet cryptococcosis continues in resource-limited settings due to limited access to ART and failure of retention in care [7]. Asymptomatic, subclinical cryptococcal antigenemia precedes meningitis by weeks to months, and has been shown to predict onset of fulminant meningitis. Cryptococcal antigen (CrAg) prevalence rates of 4–10% in persons with CD4<100 cells/µL worldwide [8]–[11], and screening for asymptomatic antigenemia followed by subsequent preemptive antifungal therapy is being undertaken in areas of high disease burden.

The new point-of-care CrAg lateral flow assay (LFA) (Immuno-Mycologics Inc, Norman, Oklahoma) has excellent diagnostic performance in CSF and serum, and good performance in urine [12]–[14]. The LFA is stable at room temperature, has a rapid turnaround time of 10 minutes, requires very little or no technical skill, and can be performed with minimal laboratory infrastructure. However, the sample types, CSF and blood, that are commonly used are obtained by invasive methods, which may be problematic in rural, primary health centres in Africa. Hence, an alternative sample type that is easily obtained may be of clinical utility. We evaluated the diagnostic performance of CrAg LFA testing of saliva compared to serum or plasma in both symptomatic and asymptomatic patient populations in Uganda. We sought to determine the applicability of saliva as an alternative sample type for cryptococcal diagnostics.

## Methods

### Study Design and Ethics Statement

We evaluated the diagnostic performance of saliva CrAg LFA testing in two prospective cohorts. The first cohort included sequential persons presenting with suspected meningitis to Mulago National Referral Hospital in Kampala, Uganda during the Cryptococcal Optimal ART Timing (COAT) trial (ClincalTrials.gov: NCT01075152) [15]. The second cohort was among those who attended the Infectious Disease Institute (IDI) outpatient HIV clinic between November 2011 and May 2013 as a sub-study of the Operational Research for Cryptococcal Antigen Screening (ORCAS) trial (ClinicalTrials.gov: NCT01535469).

Participants included in the study were ≥18 years, HIV-infected, ART naïve, with a CD4^+^ cell count <100 cells/µL and eligible to start ART. Those with a known history of previous cryptococcosis or unable to provide both saliva and a blood specimen were excluded. All research participants or their surrogate provided written informed consent. Ethical approval occurred from the Uganda National Council of Science and Technology (UNCST), Mulago Hospital Research and Ethics Committee, Makerere University Institutional Review Board, and University of Minnesota.

### Study Procedures

#### Symptomatic patients

Symptomatic adults presenting with suspected meningitis on the infectious disease ward at Mulago hospital had collection of CSF, venous blood, and saliva, after providing informed consent. CrAg LFA was performed in real time on saliva and either serum or plasma, depending on sample availability. Serum and plasma were interchangeably used in this cohort because prior validation studies we performed for the CrAg LFA showed no difference in the two samples [12].

#### Asymptomatic patients

CrAg screening was implemented among asymptomatic, ambulatory patients without signs of cryptococcal meningitis presenting to the outpatient IDI clinic. Patients were reviewed by a medical officer and assessed for pre-ART CrAg screening if their CD4+<100 cell/µL (i.e. physician-driven testing). If ART-naïve, pre-ART saliva and serum CrAg LFA test were collected prior to ART counseling. If the CrAg LFA was positive, the patient would return to see the clinician after the ART counseling session to review the results. In November 2012, the system of testing in the clinic changed to lab-based reflex testing of plasma CrAg whenever the CD4+<100 cells/µL. Any CrAg+ patient detected by lab-based reflex testing was urgently contacted and asked to return the next day for study consent. Similar procedures occurred thereafter. The clinicians received printed instructions to carefully assess the patient to rule out any signs or symptoms of meningitis, and thereafter subjects were consented for preemptive fluconazole therapy [16]. With lab-based reflex CrAg testing, the time difference between plasma and then saliva testing was 24–48 hours without any antifungal therapy. Results of serum and plasma were pooled together for both cohorts and hereafter referred to as serum/plasma or reference sample.

### Laboratory Analysis: Cryptococcal Antigen LFA Test

The LFA is a point-of-care lateral flow immunochromatographic assay “dipstick” that uses gold-conjugated, anti-cryptococcal monoclonal antibodies impregnated onto a test strip, to detect cryptococcal capsular polysaccharide antigen (i.e. CrAg) of *Cryptococcus neoformans* and *Cryptococcus gattii.* The tests were performed according to manufacturer’s instructions. Positive and negative controls were run for every set of samples. For this procedure, specimens were diluted 1∶2 in specimen diluent. For each sample, the test was carried out in duplicate. All laboratory testing was performed by two trained laboratory technologists (one for each cohort).

### Statistical Analysis

Data were analyzed using STATA version 11. Analysis established the performance of the saliva test sample in comparison to the reference sample of peripheral blood (i.e. serum or plasma). The sensitivity, specificity, positive predictive values, negative predictive values, C-statistic, and Kappa statistic for both populations were determined at 95% confidence interval (CI).

## Results

### Characteristics of the Study Population

**Figure 1:**
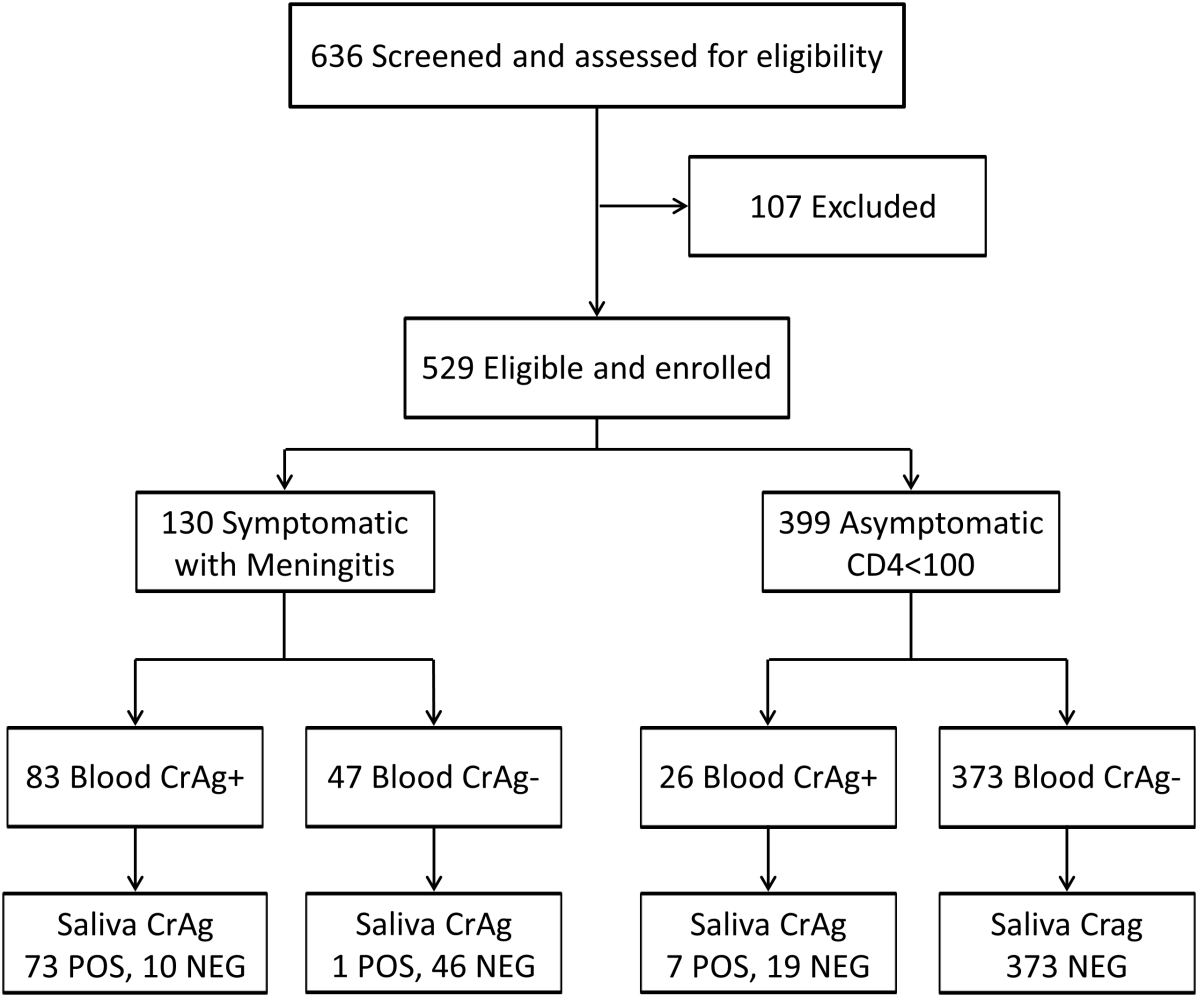
The Study enrollment and Analysis populations. The figure displays the distribution of the participants tested, consisting of HIV-infected persons from: 1) symptomatic meningitis cohort or 2) asymptomatic outpatient cohort with CD4<100 cells/µL. Saliva cryptococcal antigen (CrAg) lateral flow assay performance was compared with the reference standard of cryptococcal antigenemia in peripheral blood (i.e. CrAg+ in serum or plasma). The level of agreement between the two sample types is better in symptomatic patients (κ = 0.82) than in asymptomatic patients (κ = 0.41).

Between November 2011 and May 2013, we screened 636 potential patients with 107 being excluded for lack of paired samples. Among the 529 enrolled subjects assessed for cryptococcal antigenemia, 130 (25%) were symptomatic and 399 (75%) were asymptomatic (*Figure 1*). Both populations were similar in age and demographics (*Table 1*). The median baseline CD4+ T-cell counts among the hospitalized subjects with meningitis was 28 cells/µL (interquartile range (IQR): 7 to 70) and in the asymptomatic outpatient subjects was 38 cells/µL (IQR: 15 to 70). Overall, 81 (15.3%) saliva specimens were CrAg positive, and 109 (20.6%) serum/plasma specimens were CrAg positive.

**Table 1:** Characteristics of the study population.

Demographic Variable	Symptomatic Meningitis Cohort	Asymptomatic Outpatient Cohort
N	130	399
Women, n (%)	60 (46%)	219 (55%)
Age in years, mean (±SD)	35.7 (±9.4)	35.5 (±9.8)
CD4 cell count, median (IQR) cells/µL	28 (7–70)	38 (15–70)
CrAg positive in peripheral blood, n(%)	83 (64%)	26 (6.5%)

### Diagnostic Performance of the saliva CrAg LFA Testing

In the symptomatic population with meningitis (n = 130), the prevalence of cryptococcal antigenemia was 64% (83/130) in serum/plasma and 57% (74/130) in saliva. There were 73 true positives and 46 true negatives observed by the saliva LFA. Ten subjects (8%) had a false negative saliva CrAg, and one (0.7%) had a false positive saliva CrAg result, which was also CSF negative by CrAg and culture.

In the asymptomatic population (n = 399), the prevalence of cryptococcal antigenemia was 6.5% (26/399) in serum/plasma and only 1.8% (7/399) in saliva. There were 7 true positives and 373 true negatives observed in saliva with reference to serum/plasma. There were a substantial number of false negatives samples (n = 19, 73%), and there were no false positives registered. Specificity remained high as all serum/plasma CrAg negative patients were also negative in saliva.

Using saliva, the CrAg LFA among the symptomatic population had a sensitivity of 88% (73/83), specificity of 98% (46/47), positive predictive value of 99% (73/74) and a negative predictive value of 82% (46/56). The Kappa statistic (κ) from the comparison of saliva and serum/plasma among the symptomatic patients was 0.82 (P<0.001).

Among the asymptomatic population, saliva CrAg yielded a sensitivity of only 27% (7/26), specificity of 100% (373/373), positive predictive value of 100% (7/7) and negative predictive value of 95% (373/392). The Kappa statistic comparing saliva and serum/plasma among the asymptomatic patients was 0.41 (P<0.001).

The area under the receiver-operator characteristic (ROC) curve for sensitivity and specificity (i.e. C_statistic_) was 0.93 (95% CI: 0.888–0.970; *P* = 0.021) in the symptomatic population and C_statistic_ = 0.64 (95% CI: 0.547–0.721; *P* = 0.044) in the asymptomatic population (Table 2). The inter-assay reproducibility was good as there were no observed discordant LFAs when saliva LFAs were tested in duplicate.

**Table 2:** Summary of diagnostic performance of Cryptococcal Antigen LFA on saliva using serum/plasma as the reference standard.

Population	Sensitivity	Specificity	PPV	NPV	C-Statistic	Kappa
**Symptomatic meningitis Cohort**	88% (73/83)	97.8% (46/47)	98.6% (73/74)	82.1% (46/56)	0.929 (P = 0.021)	0.82 (P<0.001)
**95% CI**	(79 to 94%)	(89 to 100%)	(93 to 100%)	(70 to 91%)	(0.888–0.970)	
**Asymptomatic Outpatient Cohort**	26.9% (7/26)	100% (373/373)	100% (7/7)	95% (373/392)	0.635 (P = 0.044)	0.41 (P<0.001)
**95% CI**	(12 to 48%)	(99 to 100%)	(59 to 100%)	(92 to 97%)	(0.547–0.721)	

Data presented are the percentage, numerator/denominator, and 95% confidence interval. PPV = Positive predictive value, NPV = negative predictive value, C- statistic = receiver-operator characteristic (ROC) area under the curve (AUC) for sensitivity and specificity.

Saliva CrAg detectability was related to antigen burden. Among the patients with true positive salvia LFA tests, the median peripheral blood CrAg titer was 1∶8192 (IQR, 1∶1800 to >1∶64,000). In contrast, among patients with false negative saliva CrAg testing, the median peripheral blood CrAg titer was 1∶80 (IQR, 20 to 700), with a statistical difference (P<0.001).

## Discussion

This study determined the diagnostic performance of testing saliva for CrAg using the LFA in a real world clinical setting. The performance of CrAg on saliva was inadequate among patients presenting with symptomatic meningitis with an imperfect sensitivity (88%) and positive predictive value (99%). Yet, CrAg testing of saliva demonstrated poor sensitivity (27%) among asymptomatic patient populations. However, the specificity and positive predictive value remained excellent for this cohort.

When testing saliva, the LFA test strip often displayed very weak bands suggestive of a lower antigen concentration in this sample. In addition, there was a significant difference observed in CrAg titers while comparing true positives verse false negatives. Patients with false negative salvia CrAg tests had lower CrAg titers in blood.

From a practical lab perspective, plasma was easier to work with than serum or saliva with more rapid turnaround time. Although the 88% sensitivity in symptomatic meningitis patients was imperfect, the sensitivity of salvia LFA testing was higher than that of India ink microscopy (85%) in the same cohort [12].

Although the sample size was robust, a limitation of the study is that there was only one sole reader of the LFAs for each cohort. The study is likely generalizable to other sub-Saharan hospitalized and clinic populations where the burden due to cryptococcosis is high. Although we assessed the potential for testing saliva as an alternative sample compared to serum/plasma, the clinical utility is not feasible. Urine or fingerstick specimens are better non-invasive bodily fluid for testing.

In this same meningitis cohort, the CrAg LFA of urine had 97% sensitivity (151/156) and 85% (68/80) specificity for cryptococcal meningitis when using meningitis as the reference standard [12]. Noting, that those with a “false” positive urine did have cryptococcal antigenemia in peripheral blood [12]. Similarly in a Cape Town cohort, urine sensitivity was 98% (61/62) among persons with a history of cryptococcal meningitis with unknown specificity (without a control group) [13].

This study demonstrated poor diagnostic performance of saliva CrAg LFA testing. Further diagnostic evaluation of alternative sample types such as urine and capillary fingerstick are needed among symptomatic and asymptomatic HIV-infected populations in resource-limited regions. Ignoring this neglected disease will not decrease its impact on vulnerable patients in resource-limited settings.

## Supporting Information

Data S1 **Full data set for both cohorts with raw data, and CrAg titres for the meningitis cohort.**
(XLSX)
